# Assembly of infectious enteroviruses depends on multiple, conserved genomic RNA-coat protein contacts

**DOI:** 10.1371/journal.ppat.1009146

**Published:** 2020-12-28

**Authors:** Rebecca Chandler-Bostock, Carlos P. Mata, Richard J. Bingham, Eric C. Dykeman, Bo Meng, Tobias J. Tuthill, David J. Rowlands, Neil A. Ranson, Reidun Twarock, Peter G. Stockley

**Affiliations:** 1 Astbury Centre for Structural Molecular Biology, Faculty of Biological Sciences, University of Leeds, Leeds, United Kingdom; 2 Department of Mathematics, University of York, York, United Kingdom; 3 Department of Biology, University of York, York, United Kingdom; 4 York Cross-disciplinary Centre for Systems Analysis, University of York, York, United Kingdom; Institut Pasteur, FRANCE

## Abstract

Picornaviruses are important viral pathogens, but despite extensive study, the assembly process of their infectious virions is still incompletely understood, preventing the development of anti-viral strategies targeting this essential part of the life cycle. We report the identification, via RNA SELEX and bioinformatics, of multiple RNA sites across the genome of a typical enterovirus, enterovirus-E (EV-E), that each have affinity for the cognate viral capsid protein (CP) capsomer. Many of these sites are evolutionarily conserved across known EV-E variants, suggesting they play essential functional roles. Cryo-electron microscopy was used to reconstruct the EV-E particle at ~2.2 Å resolution, revealing extensive density for the genomic RNA. Relaxing the imposed symmetry within the reconstructed particles reveals multiple RNA-CP contacts, a first for any picornavirus. Conservative mutagenesis of the individual RNA-contacting amino acid side chains in EV-E, many of which are conserved across the enterovirus family including poliovirus, is lethal but does not interfere with replication or translation. Anti-EV-E and anti-poliovirus aptamers share sequence similarities with sites distributed across the poliovirus genome. These data are consistent with the hypothesis that these RNA-CP contacts are RNA Packaging Signals (PSs) that play vital roles in assembly and suggest that the RNA PSs are evolutionarily conserved between pathogens within the family, augmenting the current protein-only assembly paradigm for this family of viruses.

## Author summary

Picornaviruses are important pathogens but their assembly is incompletely understood, preventing development of anti-viral drugs and vaccines. We report identification of multiple RNA stem–loops, with purine trinucleotide sequence motifs in each loop, distributed across the genomes of two enteroviruses, enterovirus-E and poliovirus. Each motif appears to have affinity for its cognate capsid protein. Cryo-EM reconstruction of enterovirus-E without imposed symmetry identifies many repeated CP-RNA contacts. These include stem-loops adjacent to the particle two-fold axes, which appear conserved between viruses, as are the amino acid side-chains that contact all the genomic RNA segments. Mutagenesis shows that these amino acids are essential for virion formation, consistent with an RNA-dependent assembly mechanism. These results suggest that all enteroviruses utilise a common assembly mechanism that requires defined CP-RNA contacts.

## Introduction

Positive-sense, single-stranded (ss)RNA viruses of the *Picornaviridae* family cause major disease in a wide range of vertebrate species and include >30 genera [1]. Enterovirus-E (EV-E), formerly known as bovine enterovirus [2], a surrogate for poliovirus (PV), belongs to one of the best studied genera, the enteroviruses, which include PV and the human rhinoviruses [3]. Although ubiquitous it causes predominately asymptomatic infections in cattle [4]. PV is the subject of a WHO-sponsored vaccination-based eradication campaign [5]. Unfortunately, it has not been possible to develop similar effective vaccines for most picornaviruses, creating unmet needs for anti-virals. Virion assembly would make an ideal target for such therapy.

**Fig 1:**
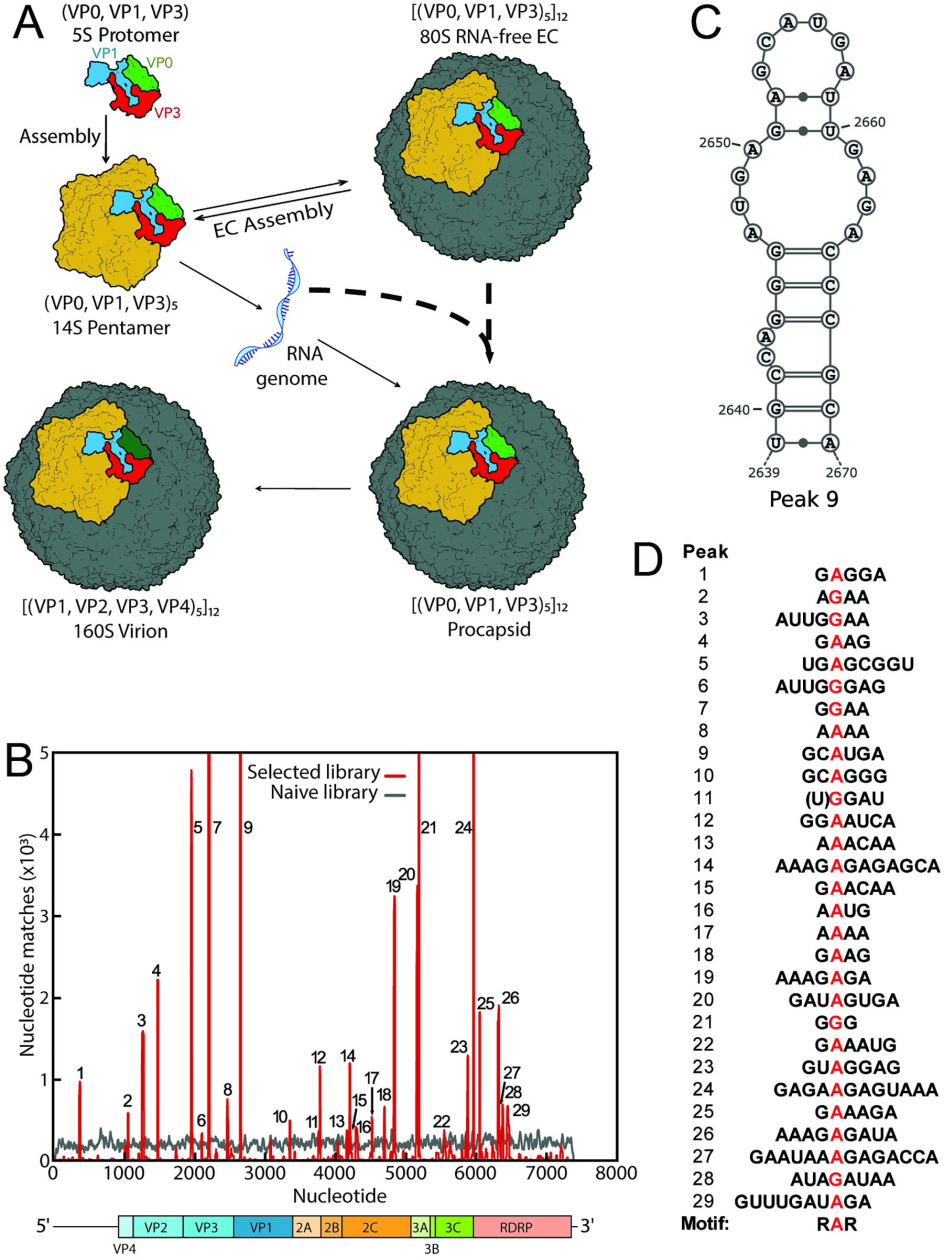
EV-E assembly pathways and SELEX against EV-E ECs. (A) Proposed enterovirus capsid assembly pathways. The viral polyprotein is cleaved by the virally-encoded 3CD protease forming protomers that rapidly self-assemble into pentamers, a proportion of which in turn self-assemble into RNA-free ECs in which VP0 subunits remain intact. It has been proposed that pentamers can interact with genomic RNA and assemble directly or enter preformed ECs, both pathways generating a procapsid. Maturation to infectious virion occurs following cleavage of VP0 into VP2 and VP4. These species are distinguishable by centrifugation and their respective sedimentation coefficients are shown. (B) Bernoulli Plot of Sequence Identities of anti-EV-E CP pentamer aptamers to the gRNA (7,414 nts long NC_001859). Each aptamer selected region was assessed for sequence matches to the genome, equivalent to 12 continuous identities, by sliding the sequence across the gRNA 5′ to 3′ incrementing 1 nt at a time. At each nucleotide position fulfilling this requirement a counter was incremented by 1 for each match, resulting in the peaks seen in red. In grey is the equivalent for the unselected starting library. Peaks 7, 9, 21, 24 have matching frequencies ~ 20, 37, 11 and 15 x10^3^, respectively, i.e. are well off the scale of the y-axis. The gene map is shown below the Bernoulli plot. (C) Mfold structure of the SL that can form from the gRNA sequence in Peak #9, the highest peak in (B). (D) Alignment of loop motif regions identified from SELEX peaks. A common RAR motif is found.

Picornaviruses assemble to form icosahedral particles with pseudo *T* = 3 symmetry, enclosing a single copy of the genome, gRNA, ~7-10k nts long [1]. Despite extensive investigation [6–12] the assembly mechanism for these viruses is still poorly understood. These virions are formed from twelve ~14S pentameric capsomers, formed of five copies of a virally-encoded polyprotein. The polyprotein is processed by a virally-encoded protease into VP0, VP3 and VP1. VP0 is cleaved into VP2 and VP4 at a late stage in assembly and maturation in most picornaviruses. VPs 1–3 form the outer surface of the viral capsid with VP1 located around the five-fold axes, and VP2 and VP3 alternating around two- and three-fold axes. VP4 is internal to this shell. Fig 1A shows a schematic of proposed virion assembly pathways [13–16]. Capsid protein (CP) pentamers also form empty ~80S protein shells *in vivo* (empty capsids: ECs) that may serve as intermediates in assembly of mature 160S virions by internalisation of gRNA [13, 15]. A more plausible alternative is for pentamers, which are known to be able to bind RNA unlike ECs [16], to bind the genome directly assembling a closed shell around it [14,17]. There is some evidence suggesting that the initial particle formed is a “pro-virion” in which VP0 remains intact. Nucleotide bases at many sites within the genome then participate in maturation by providing the chemical bases required for the proteolytic cleavages of each copy of VP0 into VP2 and VP4 [18]. This hypothesis is consistent with identification of VP0 cleavage mutants adjacent to the cleavage site for PV [7,9,19,20].

We recently proposed a novel assembly mechanism for a sub-group of the *Picornaviridae*, based on human parechovirus-1 (HPeV-1) [12]. Parechoviruses A and B, e.g. HPeV-1 and Ljungan virus, differ from typical enteroviruses because their VP0 subunits never undergo cleavage. They are also unusual because all virion structures to date contain ordered regions of density corresponding to portions of their gRNAs in contact with the interior CP surfaces at each five-fold vertex [12,21–24]. In contrast, very little ordered genomic electron density is seen in the many enterovirus structures to date, including EV-E [3] and EV-F, which cleave VP0 [25]. We showed that the RNA fragments in the HPeV-1 genome are recognised by its CP sequence-specifically, and function as RNA Packaging Signals (PSs) [12]. PSs have been extensively characterised in other positive-sense, ssRNA viruses as cognate CP-binding RNA motifs, dispersed across gRNA that facilitate co-operative, efficient and highly selective RNA encapsidation [26–30].

Here we have re-examined the structure and assembly of mature enteroviruses using EV-E and PV. Using pulse-chase experiments with EV-E, we confirmed that the VP0-containing CP pentamers within ECs can be recycled *in vivo* into mature virions. RNA SELEX was used to isolate preferred RNA-binding motifs (aptamers) for such pentamers. Alignment of the aptamer sequences against the EV-E genome, and its strain variants, identifies many putative PS sites, i.e. sequences that are likely to have affinity for pentamers. A similar result is seen for PV. We also used cryo-electron microscopy (cryo-EM) to solve the structure of EV-E to the highest resolution to date, revealing extensive density we ascribe to gRNA within the CP shell. Although this density is at lower resolution than the CPs, it contains a number of high resolution features at sites of CP-gRNA interaction. Mutational analysis of the CP side-chains confirms their importance. A subset of these occur at two-fold axes, hinting at their role in assembly. There are extensive similarities with PV, and the protein PS-binding sites appear widely conserved across the enteroviruses. These data suggest both that picornaviruses belonging to different genera use a common PS-mediated assembly mechanism and that its components are evolutionarily related.

## Results

### EV-E ECs are a source of assembly competent pentamers *in vivo*

Previously, EV-E structural proteins were over-expressed in insect cells and purified *in vitro* as ECs at high ionic strength (1 M NaCl). Those ECs, built from CP pentamers, do not disassemble when the ionic strength is lowered, leading to the suggestion that they are likely “dead-end” products off-pathway to virion assembly [15]. Since ECs are common in many picornaviral infections, we decided to investigate this idea further. ECs were accumulated *in vivo* by treating EV-E-infected cells with guanidine hydrochloride (GuHCl) to inhibit viral RNA replication whilst allowing protein translation to continue [31]. ECs and mature virions are readily distinguished by negative-staining by electron microscopy and can be separated on sucrose density gradients. The ratios of radiolabelled ECs:virions were assessed over time (S1A Fig). As expected post-GuHCl addition, the amounts of labelled EC increase with concomitant falls in labelled virions. When these cells are switched to guanidine-free (and radiolabel-free) media releasing the block on RNA replication, there is a progressive transfer of radiolabel from EC to virion fractions, in which maturation of VP0 into VP2 and VP4 occurs (S1B Fig). This result is consistent with ECs acting as stores for CPs that subsequently assemble into virions, and therefore that VP0-containing pentamers interact with gRNA.

### Unprocessed EV-E pentamers have sequence-specificity for binding RNA

RNA SELEX against CP capsomers is an effective way to identify the RNA PSs within cognate gRNAs [32]. To identify putative PS sites in the EV-E gRNA, we carried out SELEX against VP0-containing EV-E pentamers (S1C Fig). Biotinylated ECs were immobilised on streptavidin-coated magnetic beads (Materials and methods), then dissociated by raising the pH to 8.5, conditions that we have confirmed dissociate non-immobilised samples to pentamers (not shown). This yields bead-immobilised, VP0-containing pentamers orientated such that the pentamer interface proximal to the gRNA in a virion is outward-facing. These targets were exposed to a naïve N40 SELEX RNA library, allowing sequences with affinity for the pentamers to bind (S1D Fig). At each SELEX round, beads carrying pentamers and their bound aptamers were collected magnetically and unbound RNAs washed away. The bound aptamers were recovered by heat denaturation and this n^th^ round pool used as a substrate in RT-PCR reactions creating a DNA template for production of the next RNA selection pool. After 10 rounds, the recovered aptamer pool was used to prepare cDNA, which was NextGen sequenced, resulting in ~2.5 million sequence reads containing the expected primers within an oligonucleotide 110 nts long. Many selected aptamer sequences occur >20k times, compared to a maximum of just 8 times for the most frequent sequence in the naïve library (S2A Fig). The base composition of the selected library also differs significantly from both the naïve library (S2B Fig), and the EV-E genome (not shown). All of these outcomes are indications of successful selection.

Each selected unique aptamer sequence was scanned against the EV-E genome sequence (NC_001859) to identify regions of statistically significant similarity, i.e. putative RNA PSs (S1 Data). Multiple RNA sequences can be functionally equivalent in protein affinity, e.g. varying at only a single base or base pair. Detailed analysis (Materials and methods) suggests that the majority of the final SELEX pool contain sequences with characteristic folds and loop sequences. However, in order to identify the important features of the genomic matches we refine this pool, using only the ~2% of aptamers with matches statistically equivalent to that of 12 identical nucleotides in a row, i.e. 1 in ~17 million. Fig 1B shows the resulting Bernoulli Plots for both the naïve and selected libraries. There are 29 peaks (red) across the gRNA resulting from the matching of multiple individual aptamers, mostly formed from matches within the top one hundred most frequent sequences within the selected library. These match much more frequently than the highest background peak in the naïve library (grey, see also Materials and methods). As a further control, aptamers selected against both MS2 CP match the EV-E genome no more than the random starting library (not shown). Matches occur throughout the gRNA with the exceptions of VP4, 3B and the short 3´ UTR region (S2C Fig). Genomic regions corresponding to these matches most likely encompass motifs with cognate affinity for CP pentamers. They are numbered sequentially from the 5´ end of the gRNA, with Peak #9 being the most frequently matched (Fig 1B). The aptamer sequences were also scanned across the sequences of the other 14 known complete EV-E strain variants, revealing similar numbers of matching peaks in each case. Strikingly, there is extensive conservation of putative PS locations (±10 nts) with the parent strain used here, ranging from 87% at Peak #29 down to 7% for Peaks 1, 6 and 16 (S2C Fig). Similar analysis of a reference EV-F strain (S2D Fig) reveals 12 peaks that are coincident with those found in the EV-E gRNA, and an additional 10 peaks (denoted by letters) that are not. Scanning the other 10 complete EV-F strain sequences reveals that these sites are also extensively conserved (S2E Fig). Randomised reshuffling of PS peaks (1000x) yields an average peak conservation of ~20%, implying that the above conservations are significant (Materials and methods). These results are consistent with the identification of multiple, functionally-important gRNA sites, as would be expected for virions that use a PS-mediated assembly mechanism.

Analogous SELEX with an N30 library against a VP0-containing pentamer from PV Mahoney (S1E and S1F Fig) yielded aptamers that were sequenced prior to the NextGen era and that match sites across that gRNA [33]. These gRNA sequences have features reminiscent of those identified by the anti-EV-E library in the EV-E genome. We therefore used the additional statistical power of the anti-EV-E library to search for matches within the Mahoney gRNA, identifying 37 matching peaks (S2G Fig), 9 of which are coincident with the sites matched by the anti-PV aptamers, see below.

### A specific CP-binding motif

The sequences of the putative PSs from the Bernoulli plot for EV-E (NC_001859) gRNA were then compared, in order to identify any common feature(s) that would explain their pentamer binding potential. In contrast to the situation in HPeV-1 [12] there are no extended, shared sequence motifs. However, PS motifs in some viral families are known to be extraordinarily sparse, being as small as two purine nucleotides at the start and end of a tetraloop within the STNV-1 genome [30]. In the EV-E Bernoulli Plot, 14 of the 29 peaks above background match >1000 aptamer sequences, suggesting that the essential CP-binding features are shared by these genomic sites. Of these, 13 sequences are predicted by Mfold [34,35] to be stem-loops (SLs) with purine-rich loops as the lowest free energy structures, and the 14th has a similar structure as its second lowest free energy conformation. Further analysis of their tertiary structures via RNAComposer [36] reveals that all have loops in which a single-stranded purine, predominantly an adenine, is the most distal base from the top of the base-paired stem (Figs 1D and 3I). Extension of this analysis shows that all 29 putative PS sites are capable of forming the same structure. Alignment of their loop sequences based on the “A” identifies a purine bias of 75%R 100%R 93%R in the trinucleotide loop motifs, with the most frequent being GAG.

Similar analysis of the sites matched within the gRNAs of PV Mahoney and the EV-F reference strain reveals very similar motifs with slight preference changes for the dominant purines within the motif, being GAA for EV-F and AAR for PV (S2F and S2H Fig).

### Cryo-EM reconstruction of EV-E reveals extensive RNA density and multiple gRNA-CP interactions

Crystal structures for both EV-E [3] and EV-F [25] have been reported, but reveal little to no gRNA density in each case, reflecting the situation in all other picornaviral structures to date. Virus structure determination, however, has recently been revolutionised due to developments in cryo-EM. In contrast to crystallography, EM records images of individual viral particles allowing subsequent structure determination without the intrinsic averaging enforced by crystallisation. Modern software also allows refined searches for significant asymmetric density, dramatically altering our understanding of viral structures, which have previously been subjected to symmetry averaging [37–41]. This reveals limitations of previous structure determinations, and allows identification of almost complete genomes in some cases [39,42,43]. We therefore carried out cryo-EM structure determination for EV-E in the hope that the RNA-CP contacts inferred from the SELEX experiments would be visible.

### The atomic structure of the EV-E virion from cryo-EM

**Fig 2:**
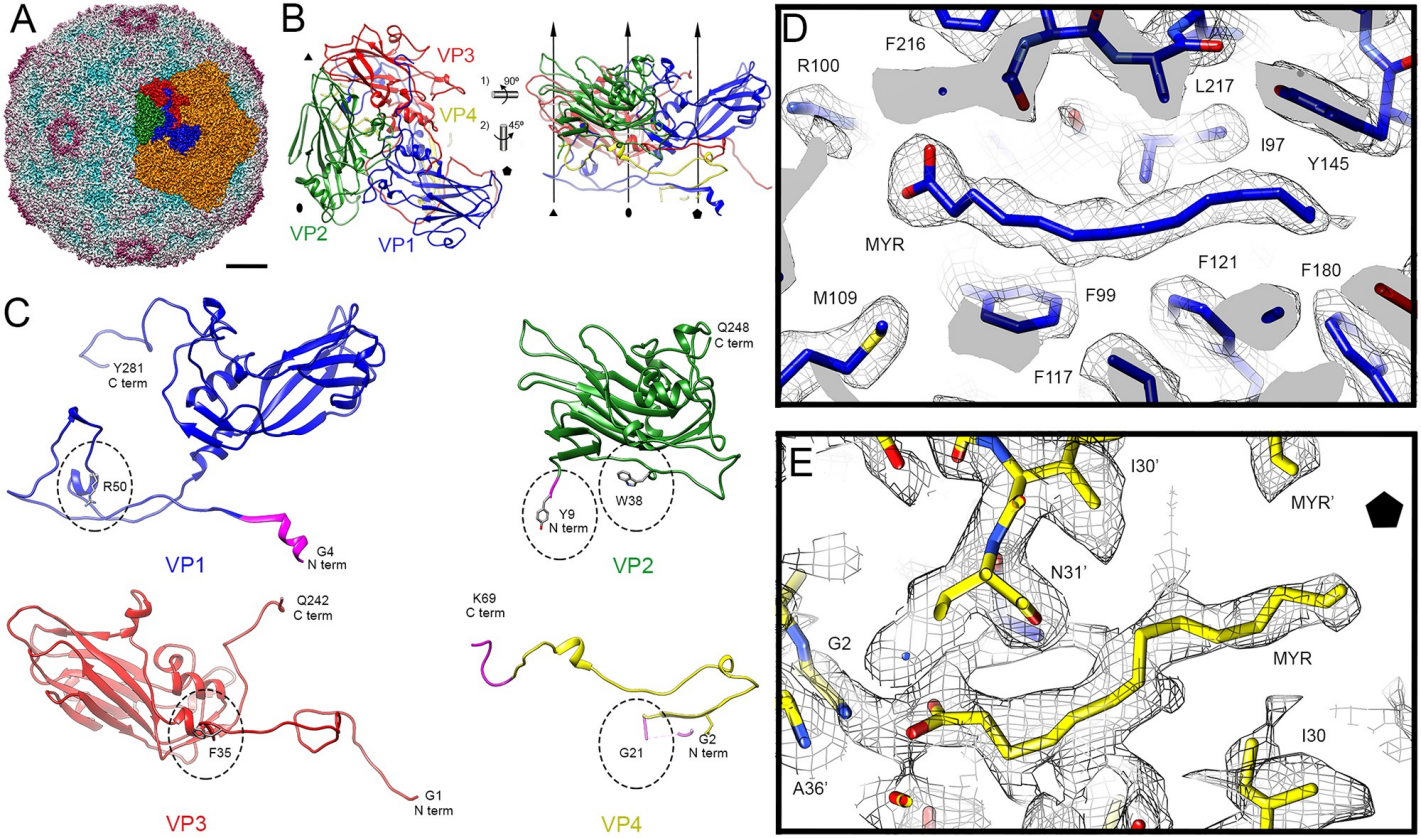
Cryo-EM 3D reconstruction of EV-E virions at 2.2 Å resolution. (A) Radially colour-coded cryo-EM density map of EV-E capsid shown at 2 σ and viewed along a two-fold axis. One of the pentamers is coloured in orange and the asymmetric unit is coloured following the “classic” colour-code for picornaviruses, VP1 in blue, VP2 in green, VP3 in red and VP4 in yellow. Bar = 50 Å. (B) Atomic model of the asymmetric unit of EV-E shown as ribbon diagrams (top view, left; side view, right) colour-coded as in (A). Symbols and arrows indicate icosahedral symmetry axes. (C) Atomic model of EV-E viral proteins shown as ribbon diagrams and colour-coded as previously. First and last residues at N- and C- termini are indicated. Amino acid side chains for residues involved in contact with gRNA are shown as heteroatom stick diagrams and indicated by dotted circles. Updated atomic coordinates for VP1, VP2 and VP4 are coloured in pink. (D) VP1 pocket factor and (E) VP4 N-terminal domain densities filled by myristic acid molecules shown as stick diagrams and colour-coded as previously, fitted into the 2.2 Å resolution cryo-EM density map shown as grey mesh. Residues are indicated and coloured by heteroatom.

The structure of the EV-E virion was determined by cryo-EM single particle analysis at 2.2 Å resolution (Fig 2 and S3B–S3H Fig). As expected [3], the capsid has minimum and maximum diameters of ~21 nm and ~32 nm, respectively. Atomic models of the four capsid proteins reveal typical picornavirus-like folds, having jelly-roll motifs with extended N-terminal arms that are not all complete within the map. The cryo-EM density allows us to build atomic models for more of these features than was possible in the previous X-ray map. The atomic coordinates for VP1, VP2 and VP4 (Fig 2C) were updated from the X-ray structure and ~20% of the previously missing residues were unequivocally assigned (Fig 2C, regions in pink). The VP1 N-terminal domain was updated with 10 additional residues, which extend to the innermost surface of the five-fold pores. The previous map made discrimination of the VP2 N-terminal and the C-terminal domain of VP4 difficult. The structure in this region was corrected and modelled according to the EM density map, which is similar to that of the recently determined EV-F X-ray structure [25]. This density map also allowed us to assign the VP2 sequence up to residue Y9, which is located precisely at the three-fold axis. VP4 was assigned up to residue G21 (orientated towards the five-fold axis) and its C-terminus completed up to K69. The VP1-VP2-VP3 junctions result in depressions known as canyons, thought to be the attachment sites for cellular receptors [44].

There are two myristic acid groups associated with the structure. One is bound as the VP1 pocket factor. Its density fits perfectly for a myristic acid molecule in a linear conformation (Fig 2D), and these sites seem fully occupied. A second myristate is visible at the end of a disconnected portion of protein density, which we assigned to the G2-Q4 position of VP4 both because of its location with respect to the remainder of VP4 and the known myristylation site at the N-terminus of poliovirus VP4 [45] (Fig 2E).

### Contacts between EV-E CP and RNA genome

**Fig 3:**
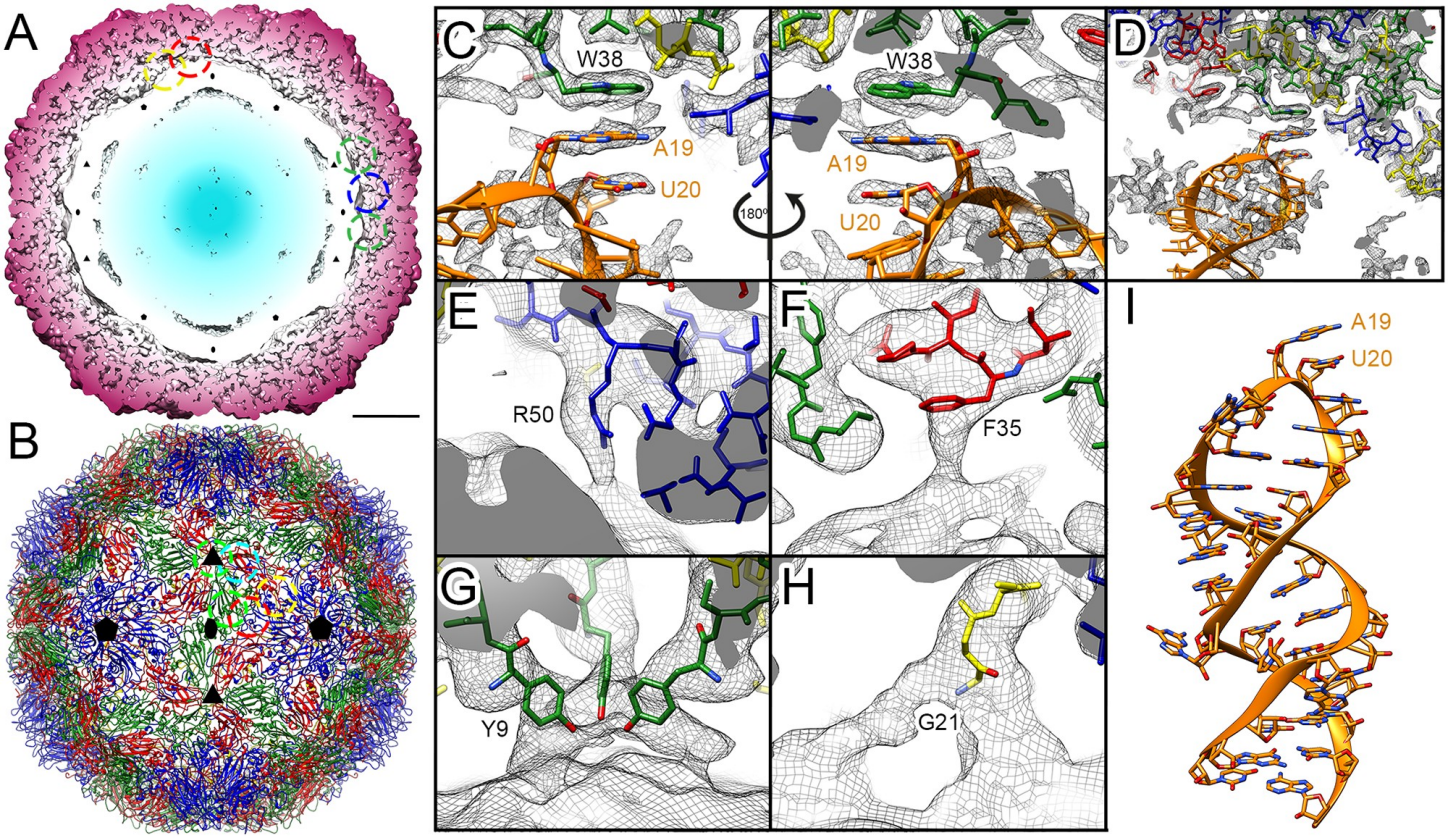
Contacts between CP and gRNA in EV-E. (A) Radially colour-coded slab of the cryo-EM unsharpened density map of EV-E capsid shown at 1.6 σ and viewed along a two-fold axis. Dashed circles indicate contacts between CP (dark red) and gRNA (white and blue). Symbols indicate icosahedral symmetry axes. Bar = 50 Å. (B) Atomic model of the front half capsid of EV-E shown as ribbon diagrams, colour-coded as in Fig 2 and viewed along a two-fold axis. Colour-coded dotted circles indicate location of contacts between CP and gRNA on the capsid. (C-H) Detail of the contacts between CP and gRNA indicated in (A, B). The cryo-EM density maps are shown as grey mesh with the density corresponding to RNA facing the bottom part. The atomic model is shown as ribbon diagrams and sticks coloured in orange for the RNA, and sticks colour-coded as previously for the CP. Residues involved in the contact are indicated and coloured by heteroatom. (C) Base stacking contact close to two-fold axis (green dotted circle in A, B) between VP2 W38 and A19-U20 bases of the RNA model generated for the most frequent PS in EV-E (Fig 1B), fitted into a local resolution low-pass filtered density map obtained after symmetry expansion and focused classification on two-fold axes shown at 1.8 σ. (D) Zoom out view of (C) showing fitting of the RNA model into lower resolution density adjacent to the contact. (E) Contact between VP1 R50 and RNA close to two-fold axis (blue dashed circle in A, B) fitted into the same map as in (C) but low-pass filtered to 5 Å resolution shown at 1.3 σ. (F) Contact between VP3 F35 and RNA close to two-fold axis (red dashed circle in A, B) fitted into the icosahedrally averaged map low-pass filtered to 5 Å resolution shown at 1.5 σ. (G) Contact located on the three-fold axis (green dashed circle in A, B) involving VP2 Y9 from three different asymmetric units fitted into the icosahedrally-averaged unsharpened map shown at 1.5 σ. (H) Contact between VP4 G21 and RNA close to five-fold axis (yellow dashed circle in A, B) fitted into the same map as in (G). (I) 3D model of the lowest energy fold for Peak #9 of the Bernoulli Plot obtained by structure prediction in RNA composer shown as in (C, D).

Intermolecular contacts between CP and gRNA are clearly visible in the icosahedrally-averaged, unsharpened cryo-EM density map but not in the sharpened map, confirming the flexible and asymmetric nature intrinsic to these regions. They occur close to the icosahedral symmetry axes (Fig 3A and S1 Movie). In order to elucidate further details, the symmetry of the icosahedrally-averaged particles was expanded such that each particle was assigned 60 redundant orientations, and then subjected to 3D classification without symmetry [46]. We focused independently on the different icosahedral symmetry axes (Fig 3B), as well as the complete genome density. The different maps allowed us to solve these contacts in an asymmetric context (Fig 3C–3H).

The highest resolution contact observed is located near the two-fold axes, where the side-chain of W38 of VP2 stacks onto a nucleotide base, which is most likely a purine (Fig 3C) in the loop of a gRNA stem-loop, consistent with the sequence motif identified by SELEX. The analysis was carried out by dividing the data into ten classes that were roughly equally populated (S3I Fig). The density in each class varies, but most classes describe variations of the same density for a base stacked onto W38. This suggests that the majority (but not all) of the 60 VP2-W38 residues in the capsid have a base stacked beneath them, but that a range of precise binding modes are accommodated as would be expected for contacts to RNA PSs differing in nucleotide sequence. A 3D model [36] of the most frequently matched peak in the Bernoulli Plot, #9 (Fig 3I) can be mostly fitted as a rigid body into a low resolution density adjacent to the contact, found in a local-resolution filtered map (Fig 3D). Motif A19-20 fits easily into the high resolution density of the contact, consistent with it being one such gRNA interaction partner.

The VP2 W38 residue is widely conserved amongst enteroviruses (S3N and S3O Fig) and is often found associated with a stacked, ordered fragment of gRNA density in picornaviral structures, implying that there could be evolutionary conservation of PS contacts between viruses. An equivalent interaction with a single RNA base is seen in the icosahedrally-averaged X-ray map of the Human Rhinovirus-2 [47].

Additional lower resolution CP-gRNA contacts occur near the two-fold axes, including via the side chains of residues R50 and F35 of VP1 and VP3, respectively (Fig 3E and 3F). Further gRNA-CP contacts occur with residues within the N-terminal domains from both VP2 and VP4. One is exactly positioned at the three-fold axis and comprises the Y9 sidechains of VP2 from three different asymmetric units (Fig 3G). The other, involving G21 of VP4, is near the five-fold axis (Fig 3H).

### Functional analysis of the CP-RNA contacts

**Fig 4:**
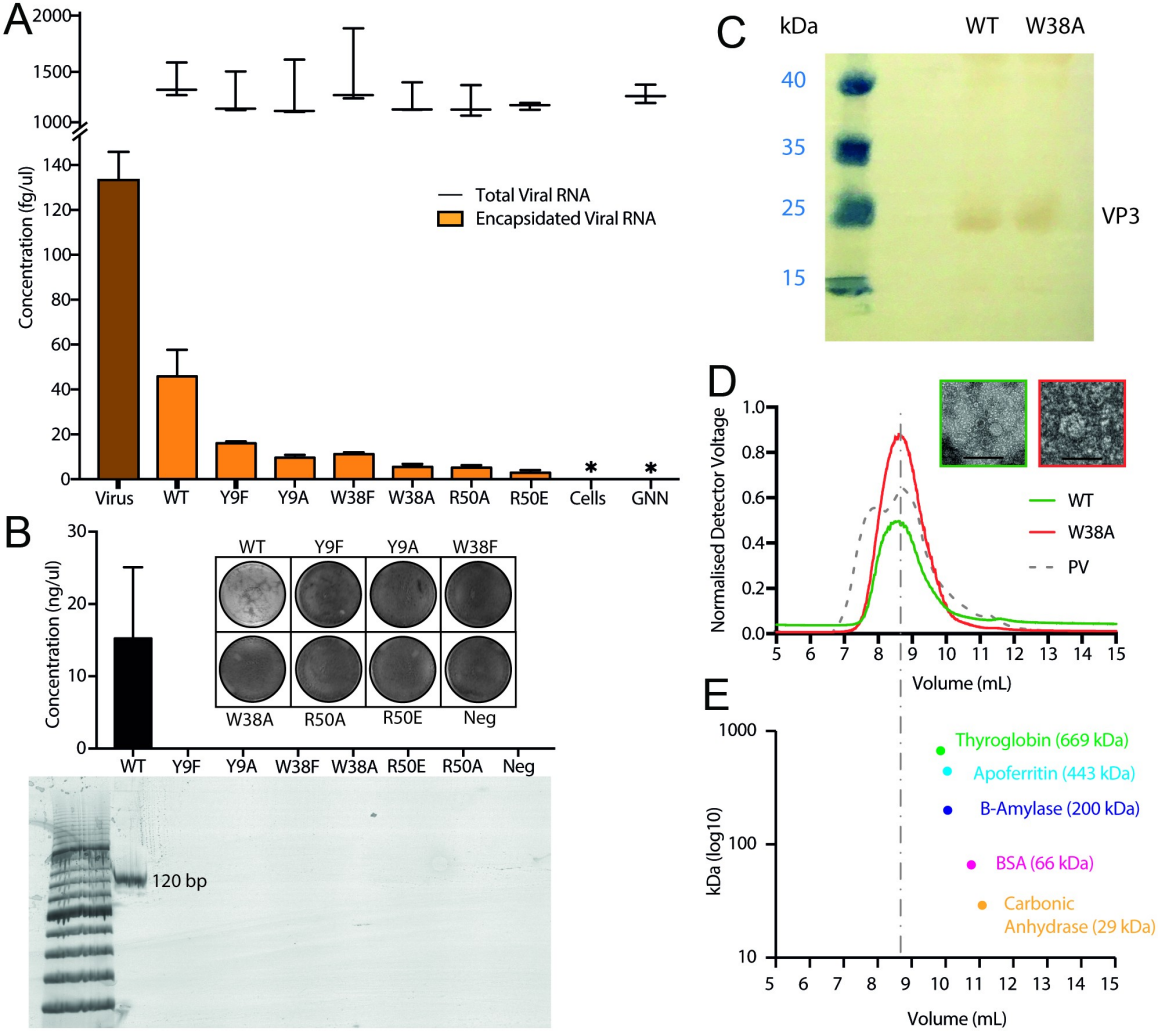
Reverse genetic analysis of the roles played by contacting CP side chains in virion assembly. (A) Wild-type and mutant EV-E genome concentrations following transfection, quantified by qPCR of region 6,981–7,081 nts. Orange bars show encapsidated viral RNA concentrations, black lines show total viral RNA concentrations. The dark orange bar labelled virus is EV-E virion infection (using virus concentration equivalent to transfections), Cells refers to cell only control, GNN is EV-E mutant with inactivated replicase. (B) Plaque assays of transfection products. Quantification of three times-passaged EV-E transfection products by plaque assays and qPCR. Following qPCR, the products were run on a gel to confirm the expected length. Lanes on gel were loaded in the order from qPCR graph; only WT showed a band which corresponds with only WT being above detectable limit on qPCR, and only WT producing plaques. (C) Western blot of WT and W38A plasmid transfection lysate probed with anti-VP3 EV-E, stained with Western Blue. (D) Light-scattering of WT (green) and W38A (red) concentrated lysate with poliovirus (PV) ECs as reference (grey, dashed line) fractionated on a TSK 6000 column in PBS. EM of WT and W38A peak fractions, scale bar represents 100 nm. (E) Protein standards run on the same column and conditions as (D) showing a plot of elution points (mL) versus known molecular weight (kDa); vertical dashed line represents the peak elution point of ECs.

In order to determine the importance of these multiple gRNA-CP contacts for virion assembly/stability, we carried out functional analysis by creating single-site amino acid changes. A reverse genetic system was established in which infectious virions are rescued from cells transfected with plasmids containing a cDNA copy of the viral genome. At each gRNA-interacting amino acid, conservative substitutions were encoded by altering one or two nucleotides within the codon in a wild-type background, allowing T7 RNA polymerase transcription to generate full-length gRNAs. Virus recovery from these transcripts followed routine protocols (Materials and methods and S4 Fig). It seems highly unlikely that such minimal perturbations would alter the ability of the 7414 nt long genome to present multiple PSs to the protein shell. Deficits in assembly and gRNA encapsidation in such modified viruses are therefore likely to arise from the elimination of important RNA-CP contacts throughout the virion. Viral replication and assembly was initiated by transfection of plasmids encoding wild-type and modified viral genomes into cells. Packaged RNA is protected from ribonuclease, enabling the total amount of encapsidated gRNA to be determined by quantitative RT-PCR (Fig 4A).

All point mutations are deleterious for gRNA encapsidation, confirming that both types of contacts seen in the cryo-EM map are functionally important. Replacement of Y9 in VP2 by F results in a >3-fold drop in encapsidation, whilst the less conservative substitution by A leads to further falls in encapsidation. Removal of the positive charge at residue R50 of VP1 (R50A) produced a ~10-fold drop in encapsidation, which is worsened by substituting the opposite charge (R50E). Even conservative substitution of the W38 residue of VP2 with the aromatic ring of F drops encapsidation 4–5 fold, and in the less-conservative A variant it is ~10-fold reduced. This is consistent with the recognition of a PS at the two-fold axes (Fig 3D). Similar assays using a viral genome with an active-site mutation to inactivate the polymerase yielded no detectable assembly/gRNA protection for the wild-type genome, implying that the limited protection with the variants is a result of nascent gRNAs being encapsidated. Western blotting (Fig 4C) confirms that the W38A mutant CP expresses similarly to wild-type. Futhermore, concentration of the lysates from these cells followed by size-exclusion chromatography, monitored by light-scattering (Fig 4D and 4E), reveals high molecular weight species in both cases. For the wild-type this material contains what appears to be both infectious virions and ECs. In contrast, no normal particles are present for the W38A variant, rather larger, apparently incomplete virus-like particles are seen by EM. The implication is that the variant CP pentamers can still assemble but fail to do so with the same geometry as the virion without the W38 contact. Consistent with these conclusions, none of the CP variants produce progeny or escape mutations after 3 passages of transfection products in Hela cells (Fig 4B), whereas infectious virions are recovered from the wild-type transfections.

## Discussion

CP-gRNA contacts are seen in cryo-EM and X-ray density maps of HPeV1, and other members of the parechoviruses [12,22,23,48] as highly-ordered genomic fragments in a ring around the five-fold vertices interacting with the inner surfaces of the viral CP pentamers. RNA SELEX against the HPeV1 CP pentamer identified a prevalent sequence motif (5´-GxUxxx-3´) that occurs at multiple, conserved sites across its gRNA. Substituting this sequence into the cryo-EM density for RNA reveals that these interactions are sequence-specific, consistent with amino acid side-chain and PS mutation. In contrast, previous structures of picornaviruses show little if any density for gRNA in contact with their CPs. In addition, it is possible to transpose non-viral genes, or extensively synonymously recode picornaviral genomes such as PV [17], with little impact on encapsidation. Such results have been used to argue that RNA motifs are not of major importance in picornavirus assembly [49].

Our results challenge that paradigm. Using anti-CP RNA SELEX [12,32,50,51] and bioinformatics we have been able to define putative PSs in both EV-E and PV, and by extension EV-F. Cryo-EM image processing of the EV-E structure permits investigation of asymmetric features within virions [40,46,52–56]. It reveals extensive relatively low resolution RNA density throughout the particle, as well as multiple, 30- and 60-fold, discrete contacts between highly-ordered sections of the gRNA and the CP shell. These gRNA-CP contacts appear to be of two distinct classes, each essential for infectious virion formation. Their protein binding sites are also extensively conserved across enteroviral structural proteins, and our data comparing EV-E PSs with those in the PV and EV-F gRNAs suggests there may be similar conservation of those sites, although there is extensive nucleotide sequence variation as expected for PS-mediated assembly. The RNA PS motifs in these viruses consist of minimalistic features (stem-loops displaying short recognition sequences). Such sites will occur frequently in many RNA sequences accounting for their robustness in mutagenesis, see below. One class of CP-gRNA contacts make relatively poorly resolved contacts to the gRNA (R50 VP1; Y9 VP2; F35 VP3 and G21 VP4), contributing to overall particle stability potentially non-sequence-specifically. A second, sequence-specific class of contacts occurs between the W38 side-chain of VP2 and an RNA stem-loop, the latter being consistent with the preferred RNA-binding motifs identified by SELEX. These are analogous to PS sites we have identified in other viral families that regulate assembly [12,27–29,50].

As we have argued for parechoviruses, there are no incompatibilities between the protein-protein mediated assembly step in which the virally-encoded 2C protein binds an incoming CP pentamer, as characterised for PV by Wimmer and colleagues [49], and an “assemblysome” in which gRNAs exiting the 2C complex, which is also a AAA+ helicase, fold locally into PSs to bind CP. The location and frequency of the 29 EV-E PSs adjacent to the particle two-folds suggests their potential role in assembly by stitching incoming pentamers into the nascent virus shell. Their sequence simplicity, a simple stem-loop with minimal purine stacking, and multiple dispersed ensemble, suggest that codon recoding or even replacement of non-structural genes would not necessarily eliminate such sites, which instead requires a detailed understanding of the nature of the PS in each case (Leonov et al., in prep.). The CP-RNA contacts characterised here can form after the gRNA emerges from the replicase-2C complex. Interestingly, the SELEX targets for both EV-E and PV were VP0-containing pentamers, yet the resulting gRNA aptamers match genomic regions in infectious virions that do not contain VP0. Clearly, these CP PS-binding sites must be accessible in both forms of the CP pentamer, and model building suggests this is the case for both viruses (S3L and S3M Fig). Although in EV-E the PSs bind adjacent to global two-fold axes, all the CP-RNA contacts made are from within a single pentamer. Indeed, these interactions may correspond to RNA sites involved in VP0 maturation as initially proposed by the Wimmer group [18].

Our results suggest that previous symmetry-averaged picornaviral structures, and the inferences drawn from them, need to be re-examined. The striking implication is that PS sites exist across the enteroviruses and that they display similar levels of conservation to the sequences of their capsid proteins. This conservation is critical, because it opens up the possibility of novel anti-viral strategies, wherein enterovirus PSs might be drug targets, whilst their manipulation within chimeric RNAs may permit the formation of protective vaccine candidates.

## Materials and methods

### Virus cultures and empty capsid (EC) production

EV-E was propagated in BHK cells, maintained in Dulbecco’s Modified Eagle Medium (DMEM, Sigma), supplemented with 10%(v/v) foetal bovine serum (FBS), penicillin (100 units/mL) and streptomycin (100 μg/mL) at 37 °C in 5%(v/v) CO_2_. BHK cell monolayers (80–90% confluency) were washed with phosphate-buffered saline (PBS) before infection with an EV-E stock (titre ~1 x 10^8^) at a multiplicity of infection (MOI) of 10 in serum-free media. The infected cells were incubated at 37°C, under 5% (v/v) CO_2_ for 8 h. Viral titre was quantified by plaque assay.

#### EC production

2 hours post-infection (hpi) the media was replaced with Met/Cys-free media (Gibco). At 2.5 hpi 10 μCi/mL [^35^S]-Met/Cys (Perkin Elmer) was added. For EC optimisation, GuHCl (2 mM final concentration) was added to media at 3, 3.5, or 4 hpi.

#### Pulse-chase

EV-E was propagated and radiolabelled as described above. GuHCl (2 mM) was added at 4 hpi to inhibit RNA synthesis and trigger EC production. At the chase time-points of 1h, 1h 15 min, 1h 30 min and 1h 45 min following GuHCl addition (corresponding to 5, 5.25, 5.5 and 5.75 hpi, respectively) the [^35^S]-Met/Cys/ GuHCl-containing media was replaced, the cells washed with PBS and the media exchanged for serum-free DMEM.

#### Virus and EC purification

EC purification was performed below 10°C to maintain native capsid antigenicity. Post-infection, lysate was freeze-thawed, transferred to a Falcon tube and 0.5%(v/v) Nonidet-P40 (NP40) added. Cellular debris was collected by low-speed centrifugation (4,000g for 10 min) and the supernatant transferred to a fresh tube. The pellet was resuspended in 0.5 mL ice-cold phosphate buffered saline, pH 7.2 (PBS) containing 0.5%(v/v) NP40, and freeze-thawed again. The solution was re-clarified by low-speed centrifugation and added to the initial supernatant. Viral particles and ECs were pelleted through a 30%(w/v) sucrose cushion at 154,000g for 5 h. The pellet was resuspended in PBS/ 0.5%(v/v) NP40 on a rocker overnight at 4°C. Virus and ECs were separated on a 15–45%(w/v) linear sucrose gradient at 354,000g for 1 h. The gradient was fractionated measuring the absorbances at A_260_ and A_280_. For EC experiments fractions were also scintillation counted. Sucrose was removed by dialysis into PBS.

#### Negative-stain electron microscopy (nsEM)

Carbon-coated copper grids (Agar Scientific, UK) were glow-discharged before application of 5 μL samples which were blotted after 30 s. Grids were stained twice with 2%(w/v) uranyl acetate and blotted dry, before viewing in a Jeol JEM-1400 TEM. Images were acquired at 40,000x magnification on a Gatan US1000 CCD camera.

### Identification of preferred gRNA motifs for CP binding by SELEX

SELEX selection was carried out largely as previously described [12].

#### SELEX target and library preparation

Purified EV-E ECs in PBS (~180 μg) were biotinylated at a 1:20 molar ratio of capsid lysines: Biotin (NHS-LC-LC-biotin). The modification was confirmed by western blotting before the ECs were immobilised on streptavidin-coated magnetic beads by rolling at 4°C overnight. Biotinylated ECs were dissociated to pentamer (as previously described [10,16]) in dissociation buffer (100 mM Tris-HCl, 3 mM MgCl_2_, 1%(v/v) NP40, pH 8.5) at 4°C for 30 min. Dissociation under these conditions was checked by nsTEM of equivalent non-immobilised samples (not shown). The beads were partitioned via magnetic separation (2 min) and washed three times in PBS to remove unbound protein. The beads were resuspended in 300 μL PBS. The naïve RNA selection library was transcribed from an N40 DNA library (TriLink Biotechnologies) (S1D Fig) using HiScribe T7 RNA synthesis kit (NEB), the DNA template was removed with Turbo DNase (Invitrogen) and purified with RNAClean XP resin (Agencourt).

#### EV-E anti-pentamer aptamer selection

The purified N40 RNA library (50 μL at 10 μM), (S1F Fig) was added to 50 μL 2× selection buffer and incubated with 20 μL of 10 mg/mL MyOne T1 Dynabeads with no immobilised protein (negative beads) for 15 min at 37°C. The beads were partitioned via magnetic separation (2 min) and the unbound fraction removed and incubated with 20 μL positive beads for 25 min at 37°C. Beads were partitioned again (2 min) and the unbound fraction removed. Beads were washed ten times (1 min incubations) with 125 μL of selection buffer to remove unbound/weakly bound RNA species before being finally resuspended in 35 μL DEPC-treated H_2_O. Bound RNA was eluted from the beads by heating to 95°C for 5 min in a PCR thermocycler, followed by magnetic removal of the beads. The released RNA was reverse transcribed and amplified using a OneTaq RT-PCR kit. The enriched library was transcribed to RNA with a T7 transcription kit, the DNA template removed with TURBO DNase and purified with RNAClean XP resin (Agencourt). The concentration of RNA was determined after each round and 1 μM RNA was added to the next round of SELEX.

CP binding stringency was increased across the ten rounds by: decreasing the positive bead incubation time over the first five rounds from 25 to 5 min; and increasing the number of washes from 10 to 15 over rounds 6–10. For rounds 5 and 10, a competition was carried out to remove RNA sequences that had a higher affinity for the EC than for the inner pentamer surface. The SELEX round was performed as above but after the washes, the positive beads were incubated with either 0.1 mg/mL of biotinylated capsid (round 5) or EC (round 10). A further three washes were carried out to remove the capsids and any capsid-bound RNA aptamers. Elution, etc, was then performed as above. The amplified DNA of round 10 was then subjected to Next-Generation sequencing using Illumina MiSeq.

#### Poliovirus pentamer SELEX

Polio pentamer SELEX followed a similar protocol to the EV-E SELEX, with differences in protocol described below. The N30 library (S1F Fig) was used to generate the RNA aptamer pool. N30 aptamers were incubated with empty beads in the absence of pentamers, RNA aptamers that did not bind to empty beads were used for the selection against the target. Counter-selections against ECs were also carried out at the 5th and 10th cycles to restrict binding species to the inside of the capsid.

The 10th round RNA pool was reverse-transcribed, PCR-amplified and ligated to the pGEM-T vector followed by transformation to *E*.*coli* supercompetent cells (Stratagene). 10 individual clones were randomly selected via blue/white screening, and then Sanger sequenced.

#### Analysis of the anti-EV-E aptamers

The Bernoulli score of a given aptamer was calculated by sliding its sequence along the genome in increments of 1 nt to generate genome sequence frames for comparison with the selected region of the aptamers. In order to treat information at the 5′ and 3′-ends of each selected region on an equal footing to the middle sections in the histogram plot, we also considered partial overlaps, obtained from alignments of the 3′ region of the aptamers to the 5′ regions of the genome and vice versa, gradually increasing in steps of 1 nt until full overlap was reached. For each such alignment, we calculated the maximum Bernoulli score, defined as *B*(*L*,*N*) = *L* − log_4_ *x* where x=∑i=0N(Li)3i [57]. This is related to the probability P(L,N)=(14)B(L,N) that a random sequence of B(L,N) letters would align precisely with the genome.

Since typically the fragment contributing to the score was smaller than the length of the aptamer and contained some mismatches, we identified the largest fragment of the aptamer that had the highest Bernoulli score, and therefore, the lowest probability of having aligned to the genome fragment by chance. For each comparison frame, the fragment of the aptamer that aligned to the genome with the maximum Bernoulli score was identified via computation of the contributions of different mismatches to the scores of any contiguous subset of the frame. If this maximum score was larger or equal to 12, we logged it into the data file (using the frequency of the aptamer read) that was subsequently used to compute the histograms used.

The library selected against EV-E capsid protein contains 2,563,876 aptamer reads, of which 147,489 are unique. 55,088 (37.35%) of the unique reads match to the genome with a matching probability equivalent to 10 nucleotides, which is ~1 in a million. In order to facilitate the identification of the PSs in the genome we used an even stricter criterion for the data shown in Fig 1B and S2A Fig and the subsequent analysis. Namely, using matching probabilities equivalent to 12 nucleotides or higher, which is ~1 in 17 million. This criterion identifies 3,013 (2%) unique reads which were used to construct the Bernoulli plots shown in the manuscript. Of these 3,013 reads, 17 match to the genome in more than one position. 15 reads match to the genome at two positions, while 2 reads match at three positions. The most frequent of these multiply-matched sequences has only 13 reads in the selected library, so their inclusion does not significantly affect the resultant histograms. For comparison, the most frequent read (above the matching threshold) has 33,238 reads in the selected library. Note, the reads from the selected library which do not match to the genome with the chosen criteria can still fold into secondary structures presenting a ‘GAG’ motif in a stem-loop. These folds are generated by folding the selected region of each aptamer with the fixed flanking sequences, which are both present in the SELEX experiment. It is a common observation that flanking sequences contribute to the functional folds of selected aptamers. These outcomes are consistent with a single dominant preferred RNA sequence, based around the PS consensus (S1 Data).

The highest peak from the Bernoulli analysis (Fig 1B, peak 9) was identified, and the genome sequence corresponding to the peak area above noise, i.e. above the curve corresponding to the alignment of the unselected library, extracted. This 19 nt long sequence was flanked by the 15 nts 5′ and 3′ to it, resulting in a 49 nt long fragment. All possible secondary structure folds (lowest energy folds) of the latter having folding energies between -4.5 to -0.3 kcal/M were determined using M-fold. The corresponding stem-loops were extracted, and compared to those obtained via the same procedure for the four next highest peaks (peak 7, 24, 21, 5), as well as the five most conserved peaks across the 15 sequences analysed (peak 29, 20, 22, 26, 14). In particular, the loop nucleotides were assessed for a common motif. In contrast to a previous analysis for HPeV1, there was no clear evidence for an extended motif, aggravated by strongly varying loop sizes, suggesting that any sequence motif would be sparse.

We therefore analysed the matches with the aptamer pool in more detail. Firstly, we note that the library was preferentially enriched for A (from 26.1% to 34.5%) and G (from 24.7% to 41.7%), whilst the genome has only a mild bias towards purines (A 26.3%; G 24.4%; C 25.0%; U 24.4%) (S2B Fig). This suggests that any PS sequences should be rich in purines. We therefore assessed the percentage of reads that occur with a frequency of more than 2 in the selected library as 93.5% (2,396,122 out of 2,563,876 total reads, including the multiplicity of reads). This is significantly higher than for the previous analysis of HPeV1 (42.9%) and HBV (43.2%), suggesting that a large variety of sequences satisfy the requirements of binding, again pointing towards a sparse recognition motif. It is therefore unlikely to find such a motif via secondary structure analysis alone.

We used RNAcomposer to render candidate PS SLs in 3D and compare with the cryo-EM density. The 3D structure of the lowest energy fold for Peak 9 is shown in Fig 3I.

### Cryo-EM structure determination

Purified EV-E samples in PBS (3 μL) were applied onto 400-mesh lacey grids coated in a 3 nm carbon film (Agar Scientific, UK). The sample was left to adsorb for 30 s before most of the sample was blotted away manually and this process was repeated 4 times. The grids were vitrified using a Leica EM GP freezing device (Leica Microsystems). Chamber conditions were set at 4 °C and 95% relative humidity. Grids were glow discharged for 30 seconds prior to application of the samples. Data were collected on a FEI Titan Krios electron microscope operated at 300 kV and images recorded on a FEI Falcon III detector operating in linear mode. A total of 8,785 movies were recorded at a calibrated magnification of 75,000x, yielding a pixel size of 1.065 Å on the specimen. Each movie comprises 39 frames with an exposure rate of 1.27 e^-^ Å^-2^ per frame, with a total exposure time of 1 s and an accumulated exposure of 49.5 e^-^ Å^-2^. Data acquisition was performed with EPU Automated Data Acquisition Software for Single Particle Analysis (ThermoFisher) at -0.75 μm to -3.5 μm defocus.

### Image processing

Movies were motion-corrected and dose-weighted with MOTIONCOR2 [58]. Aligned, dose-weighted micrographs were then used to estimate the contrast transfer function (CTF) with GCTF [59] and 8,284 micrographs were selected. All subsequent image processing steps were performed using RELION 3 [60,61]. 2D averages obtained from preliminary datasets were used as references to automatically pick the micrographs and a total of 260,348 particles were extracted. 2D classification was performed and 105,723 particles were selected to perform a 3D classification imposing icosahedral symmetry, using a model obtained from preliminary datasets low-pass filtered to 60 Å resolution as initial model. As the different 3D classes were similar, all of the particles were included in 3D auto-refinement imposing icosahedral symmetry, yielding a map with an overall resolution at 2.86 Å based on the gold-standard (FSC = 0.143) criterion. This refinement was followed by CTF refinement and Bayesian polishing [62] routines implemented in RELION 3, yielding a map with an overall resolution at 2.32 Å. Finally, this map was corrected for Ewald sphere [63] in RELION 3, yielding a final map with an overall resolution at ~2.2 Å. Local resolution was estimated using RELION’s own implementation.

Following 3D refinement with icosahedral symmetry, the symmetry of the dataset was expanded such that each particle was assigned 60 orientations that corresponded to its icosahedrally redundant views. A cylindrical mask was created to exclude all of the virion structure except the area of interest, using SPIDER [64]. We separately considered two-fold, three-fold and five-fold axes, as well as the complete genome density by masking out the entire capsid. The symmetry expanded particle dataset was subjected to focused 3D classification into 10 classes without alignment or imposition of symmetry. Particles from selected classes were 3D reconstructed and post-processed.

#### Model building and refinement

The crystal structure of EV-E [3] (PDB:1BEV) was first manually docked as a rigid body into the density and followed by real space fitting with the Fit in Map routine in UCSF Chimera [65]. A first step of real space refinement was performed in Phenix [66]. The model was then manually rebuilt in Coot [67] to optimize the fit to the density. Density corresponding to the C-terminal domain of VP4 was wrongly occupied by N-terminal residues of VP2 as in 1BEV, thus this part was corrected and built de novo. After icosahedral symmetrisation in Chimera to generate the entire capsid, a second step of real space refinement was performed in Phenix. Refinement statistics are listed in S1 Table.

#### Model validation and analysis

The FSC curve between the final model and map after post-processing in RELION (Model vs Map), is shown in S3B Fig. To perform cross-validation against overfitting, the atoms in the final atomic model were displaced by 0.5 Å in random directions using Phenix. The shifted coordinates were then refined against one of the half-maps (work set) in Phenix using the same procedure as for the refinement of the final model. The other half-map (test set) was not used in refinement for cross-validation. FSC curves of the refined shifted model against the work set (FSCwork) and against the test set (FSCtest), are shown in S3C Fig. The FSCwork and FSCtest curves are not significantly different, consistent with the absence of overfitting in the final models. The quality of the atomic model, including basic protein geometry, Ramachandran plots, and clash analysis, was assessed and validated with Coot, MolProbity [68] as implemented in Phenix, and with the Worldwide PDB (wwPDB) OneDep System (https://deposit-pdbe.wwpdb.org/deposition). Graphics were produced by UCSF Chimera [65].

### Reverse genetic assays of gRNA-CP contacts

#### Infectious EV-E clones and CP variants

The EV-E gRNA sequence (NCBI, NC_001859) was supplied by Genscript Biotech (Netherlands) in a pUC57 plasmid vector, which encompasses ampicillin-resistance. The gRNA was flanked with a 5′ T7 promoter and hammerhead ribozyme and a poly-A40 tail terminating in a unique NotI restriction site at the 3′ end (S4 Fig). Mutations to gRNA contacting amino acids were designed and were also synthesised in this vector by Genscript. These DNAs were transformed into Stable Competent *E. coli* (NEB), then extracted using a maxiprep kit (Qiagen) and purified via the Monarch DNA clean-up kit (NEB). The wild-type and mutant plasmids were digested with NotI (S4C Fig), the reactions checked on a 1% (w/v) agarose gel and cleaned up as above.

#### Transfection and viral RNA extraction

BSR-T7 cells were grown to 80% confluency in 6-well plates. Linear plasmid DNA (1 μg of each) was transfected into wells using Viafect transfection reagent (Promega). Plates were incubated for 18 h at 37°C. Post-transfection the plates were freeze-thawed to lyse cells and the media removed. The cell lysate was treated with DNase and RNase A (10 μg/mL) at 37°C for 30 min to remove plasmid DNA and non-encapsidated RNA. Encapsidated RNA was extracted using Trizol; the precipitated RNA was pelleted, washed x3 with 70% (v/v) ethanol, air dried and resuspended in 12 μL H_2_O. The RNA was DNase treated again to ensure no plasmid DNA carryover. A 1 μL aliquot was taken from each RNA for a no-RT control in the qPCR reaction. The rest of the RNA was reverse-transcribed to cDNA using a sequence-specific primer (EV-E_R_7100–7081: ACGCACGAACTTCCTCTTCA) and Superscript IV (Invitrogen).

#### Quantitative PCR

The cDNA and the no-RT controls were added to 5 μL qPCR mastermix (SensiFast SYBR, Bioline), 400 pM each of the EV-E-specific primers (EV-E_F: AGCCTCTTGGCAGAAGCTG, EV-E_R: ACGCACGAACTTCCTCTTCA), and made up to 10 μL with H_2_O. The concentration of the experimental samples was quantified using a standard curve of EV-E RNA in the same qPCR run that followed the same RT and qPCR steps as the experimental samples.

#### Plaque assay

BHK cells were grown 80% confluency in 6-well plates (Corning). Cells were washed with PBS and viral lysate from the above transfection added, made up to 1.5 mL with serum-free media (Sigma). Plates were incubated at 37°C for 2 h, then lysate was removed and the cells overlayed with 2 mL 1%(w/v) SeaPlaque agarose in DMEM with 2%(v/v) Horse Serum (Gibco) for 48 h. Cells were fixed with 2%(v/v) formaldehyde (Sigma) for 30 min, and stained with crystal violet solution (Sigma) before visualisation.

#### Passage of mutant transfections

EV-E plasmid, either wild-type (WT) or packaging signal mutation at Y9 or W38 in VP2 or R50 in VP1 was transfected into BSR-T7 cells. Following an 18h incubation, transfection product was purified by freeze-thaw and passaged three times on BHK cells. The lysate was harvested and viral titre was quantified by plaque assay and qPCR (as previously described).

#### Western blot

WT and W38A transfection product lysates were probed for EV-E VP3 by Western blot. 40 μL of each was run on an SDS-PAGE gel and transferred to nitrocellulose membrane. The primary antibody was mouse anti-VP3(EV-E) 8B1 (a gift from Paul Duprex, University of Pittsburgh, USA) diluted 1:500 in TBS-T, secondary antibody was goat anti-mouse IgG biotin (Invitrogen, 31800). The bands were visualised using Western Blue Stabilized Substrate for Alkaline Phosphatase (Promega, S3841).

#### Size-exclusion chromatography

WT and W38A lysates were pelleted through a 30%(w/v) sucrose cushion (as described above in EC purification). Lysates were loaded onto a TSK 6000 column in PBS. The A_260_ and A_280_ absorbancies, as well as light-scattering were measured. The latter are shown in Fig 4D. Size standards: Thyroglobin (669 kDa), Apoferritin (443 kDa), beta-amylase (200 kDa), BSA (66 kDa) and Carbonic Anhydrase (29 kDa) were all run on the column identically to determine their elution points (mL) as size standards.

## Supporting information

S1 Fig EV-E and PV SELEX preparation. (A) Optimisation of EV-E EC production. Analysis of viral CP radiolabelling, post-treatment with GuHCl, of an EV-E infection fractionated on a linear sucrose density gradient. Inset: nsEM images of peak fractions showing ECs and virions. EM scale bars, here and in (B), = 100 nm. (B) Pulse-chase of ECs to virions. Bars represent peak fraction scintillation counts of ECs and virions from sucrose density gradients, as in (A), following removal of GuHCl inhibition at different time points. Inset: nsEM images from peak fractions of ECs (1 h) and virions (1.75 h). (C) Confirmation of biotinylation of EV-E for SELEX target by western blot. (D) N40 library sequence information and primers for the EV-E selection. The T7 sequence is underlined in red. (E) Poliovirus EC pentamer SELEX target preparation following the same protocol as for EV-E. Sucrose density gradient fractions showing scintillation counts for each fraction. Inset are nsEM images of the peak PV virion and EC fractions and an autoradiography gel of these peak fractions. (F) N30 library sequence information and primers for the PV selection with T7 sequence underlined in red.
(TIF)

S2 Fig SELEX analysis and identification of a conserved, purine trinucleotide loop motif. (A) Frequency plot of the EV-E aptamer sequences in the selected and naïve libraries. The selected library contains 147,489 total unique reads within a total of 2,563,876 sequences (24% with a frequency >2). The most frequent aptamer read was 212,070. (B) EV-E RNA library base composition before and after selection. (C) List of peaks above background in the anti-EV-E Bernoulli Plot (Fig 1B) and their levels of conservation across all 15 fully sequenced EV-E strain variants (NC_001859.1, MG571548.1, MH719217.1, AF123432.1, MG650158.1, AF123433.1, KC667561.1, DQ092792.1, LC150009.1, DQ092793.1, DQ092769.1, DQ092771.1, KM667941.1, KU172420.1, LC081216.1). (D) Bernoulli Plot of anti-EV-E aptamers screened against the EV-F reference strain gRNA (7,397 nts long, NC_021220 gRNA) in red, naïve library in grey. (E) List of peaks above background in the EV-F Bernoulli Plot and their levels of conservation across fully sequenced EV-F strain variants (NC_021220, LC150008, DQ092795, DQ092794, LC150010, HQ917061, AY508696, HQ663846, KC748420, HQ917060, AY508697). (F) Alignment of loop motifs from EV-F sequence alignment of SELEX aptamers. Alignment of the 12 most frequently matched loop motifs reveals a preferred GAA motif. (G) N40 naïve library (grey) and EV-E selected library (red) matched against the PV Mahoney strain genome (NC_002058.3). Cognate PV SELEX peaks aligned against the Mahoney genome that are coincident with the EV-E peaks (inverted arrows for aptamers a42 in purple; a45 in orange; a50 in green). (H) Alignment of PV loop motifs, showing common AAR motif.
(TIF)

S3 Fig Details of the EV-E cryo-EM reconstruction. (A-F) Resolution and model validation of EV-E structure. (A) Cryo-EM image of EV-E. Bar = 500 Å. (B) Fourier Shell Correlation (FSC) resolution curve for the icosahedrally averaged 3DR of EV-E. Resolution based on the gold standard 0.143 criterion is 2.23 Å. (C) Cross-validation against overfitting of the model, FSC curve for the final atomic model refined against the post-processed map (green curve, Model vs Map), and FSC curves for the randomly shifted and refined atomic model against the half map used in the refinement (blue curve, FSCwork) and against the half map not used in the refinement (red curve, FSCtest). (D-F) Surface (top) and slab (bottom) viewed along a two-fold axis coloured and low-pass filtered based on local resolution as indicated in the colour key (values in Å) for the (D) icosahedrally averaged density map shown at 1 σ, (E) density map obtained after symmetry expansion and focused classification on two-fold axis shown at 2 σ, and (F) density map obtained after symmetry expansion and focused classification on genome density shown at 1.2 σ. (G-H) Quality of the cryoEM density map. G) Atomic model of the asymmetric unit of EV-E shown as ribbon diagrams (top view, left; side view, right) colour-coded as in Fig 2 fitted into the 2.2 Å resolution cryo-EM density map shown as colour-coded semi-transparent surface. Symbols indicate icosahedral symmetry axes. (H) Atomic models of EV-E viral proteins colour-coded and shown as sticks fitted into the 2.2 Å resolution cryo-EM density map shown as colour-coded mesh. Residues are indicated and coloured by heteroatom. (I) Variability of the VP2 W38-RNA contact. (I-K) Cryo-EM density maps for the 10 classes obtained after symmetry expansion and focused classification on two, three and five-fold axis shown at 2 σ and viewed as in Fig 3C, 3G and 3H, respectively. Class distribution, and CP and RNA densities are indicated. (L) EV-E VP0 processing. Atomic model of the asymmetric unit of EV-E shown as ribbon diagrams colour-coded as previously and viewed from the inner surface of the viral capsid. Symbols indicate icosahedral symmetry axes. VP2 N- and VP4 C-terminal domains are indicated. Dashed circle highlights the proximity of the VP0 cleavage site and the PS bound at the VP2 W38 contact. (M) PV VP0 processing. Atomic model of the asymmetric unit of VP0-containing PV (PDB ID: 1POV) and mature PV (1AR8) aligned to EV-E gRNA shown as in (L). VP1, VP2 and VP3 are shown transparent for clarity. Dashed black circles indicate VP0 cleavage site and VP2 W38. Yellow and green arrows indicate the structural rearrangements of the parts of VP0 that will constitute VP4 (light pink to yellow) and VP2 (purple to green). (N) Sequence alignments in EV-E and F (EV-E and EV-F). All fully sequenced genomes available in Genbank were used. Amino acids of interest, Y9 and W38 of VP2 and R50 of VP1 are highlighted. (O) Conservation of Y9, W38 and R50 within enteroviruses, using sequences P12915 (EV-E), P03303 (HRV14), P04936 (HRV2), Q82122 (HRV16), P12916 (HRV1B), P03313 (Coxsackie-B3), P03300 (PV1), Q66478 (EV71) as representatives for each virus. “*”, “:”, “.”, symbols under alignments represent perfect alignment, strong similarity and weak similarity respectively.
(TIF)

S4 Fig Functional analysis of the EV-E gRNA-CP contacts, and their conservation. (A) Schematic of EV-E infectious clone; the EV-E genome was inserted into a pUC57 vector, the structural proteins of P1 region are filled in yellow-green and the sites of interest are highlighted, green boxes denote the chosen mutagenesis sites. (B) Mutations introduced individually into the infectious clone. (C) Plasmid digestion with *Not I*; cut and uncut plasmid were run on a 1%(w/v) agarose gel to confirm digestion.
(TIF)

S1 Movie Contacts between CP and RNA in EV-E. Movie showing the reconstructed EV-E virion, together with the focused classifications at its symmetry axes. The cryo-EM density maps are shown as colour-coded surface, grey semi-transparent surface and grey mesh, with the density corresponding to RNA facing the bottom part. The atomic model is shown as ribbon diagrams and sticks coloured in orange for the RNA, and colour-coded as previously for the CP. Residues involved in the contact are indicated and coloured by heteroatom.
(MP4)

S1 Table General parameters used during Cryo-EM data collection and image processing, and general statistics obtained after refinement and validation of the model and Cryo-EM density map. (DOCX)

S1 Data Aligned histogram data for Bernoulli plots of EV-E, EV-F and polio SELEX. (ZIP)
