# Supplementary figures and images for: Assembly of infectious enteroviruses depends on multiple, conserved genomic RNA-coat protein contacts
Source: PLoS Pathog. 2020 Dec 28;16(12):e1009146. doi: 10.1371/journal.ppat.1009146 (PMC7793301; doi:10.1371/journal.ppat.1009146)

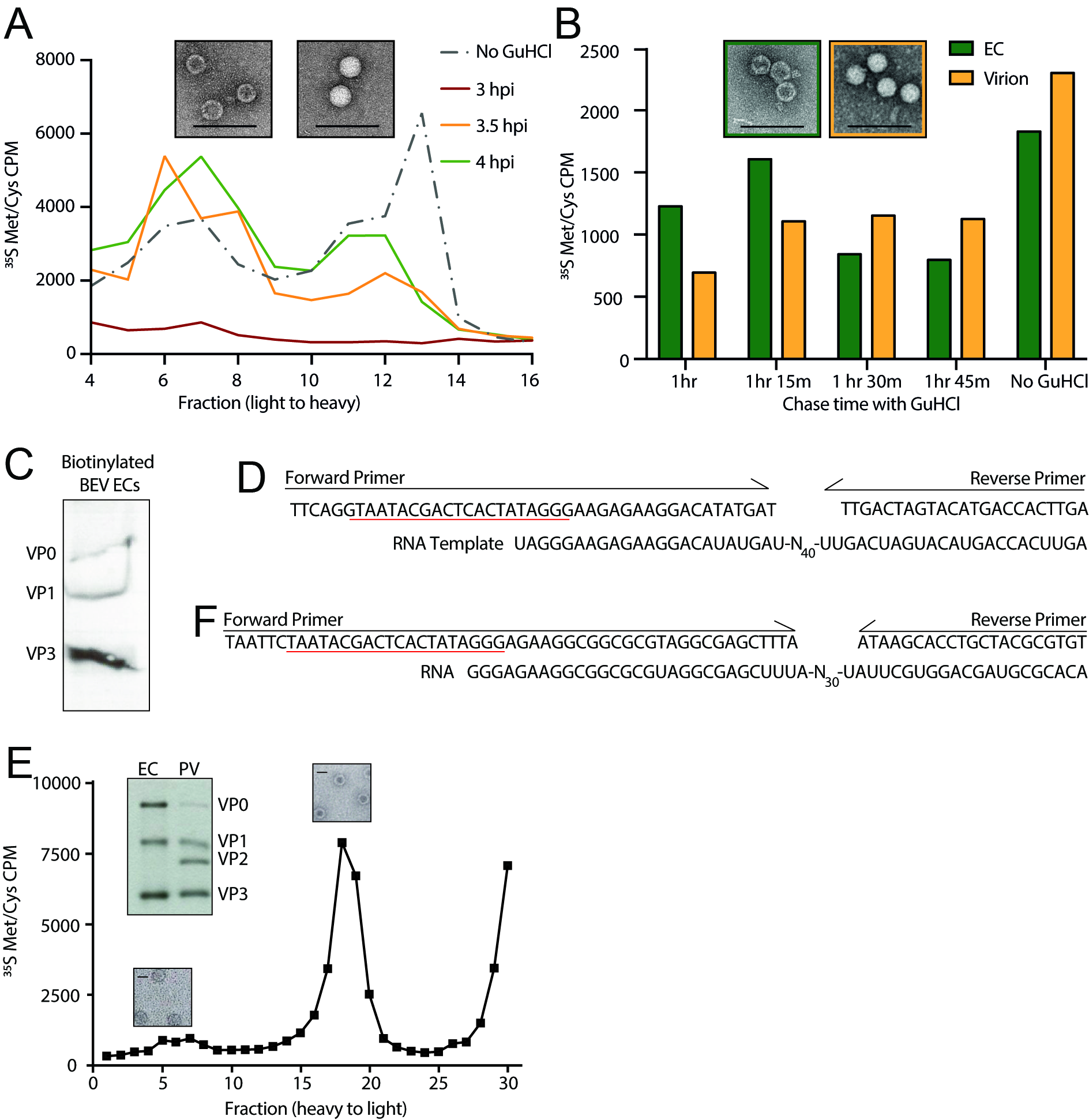

Supplement: S1 Fig — (A) Optimisation of EV-E EC production. Analysis of viral CP radiolabelling, post-treatment with GuHCl, of an EV-E infection fractionated on a linear sucrose density gradient. Inset: nsEM images of peak fractions showing ECs and virions. EM scale bars, here and in (B), = 100 nm. (B) Pulse-chase of ECs to virions. Bars represent peak fraction scintillation counts of ECs and virions from sucrose density gradients, as in (A), following removal of GuHCl inhibition at different time points. Inset: nsEM images from peak fractions of ECs (1 h) and virions (1.75 h). (C) Confirmation of biotinylation of EV-E for SELEX target by western blot. (D) N40 library sequence information and primers for the EV-E selection. The T7 sequence is underlined in red. (E) Poliovirus EC pentamer SELEX target preparation following the same protocol as for EV-E. Sucrose density gradient fractions showing scintillation counts for each fraction. Inset are nsEM images of the peak PV virion and EC fractions and an autoradiography gel of these peak fractions. (F) N30 library sequence information and primers for the PV selection with T7 sequence underlined in red. (TIF) [file ppat.1009146.s001.tif]

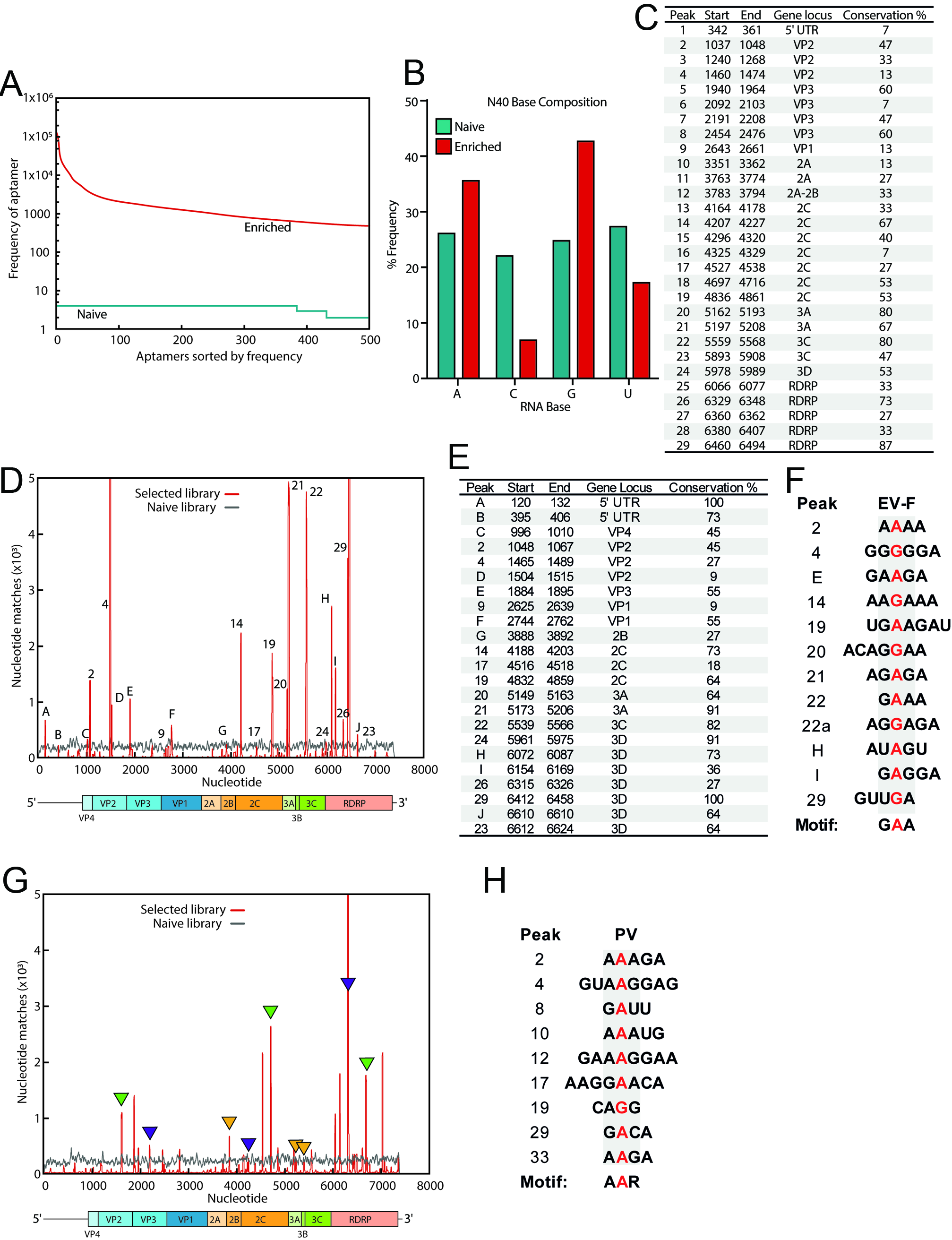

Supplement: S2 Fig — (A) Frequency plot of the EV-E aptamer sequences in the selected and naïve libraries. The selected library contains 147,489 total unique reads within a total of 2,563,876 sequences (24% with a frequency >2). The most frequent aptamer read was 212,070. (B) EV-E RNA library base composition before and after selection. (C) List of peaks above background in the anti-EV-E Bernoulli Plot (Fig 1B) and their levels of conservation across all 15 fully sequenced EV-E strain variants (NC_001859.1, MG571548.1, MH719217.1, AF123432.1, MG650158.1, AF123433.1, KC667561.1, DQ092792.1, LC150009.1, DQ092793.1, DQ092769.1, DQ092771.1, KM667941.1, KU172420.1, LC081216.1). (D) Bernoulli Plot of anti-EV-E aptamers screened against the EV-F reference strain gRNA (7,397 nts long, NC_021220 gRNA) in red, naïve library in grey. (E) List of peaks above background in the EV-F Bernoulli Plot and their levels of conservation across fully sequenced EV-F strain variants (NC_021220, LC150008, DQ092795, DQ092794, LC150010, HQ917061, AY508696, HQ663846, KC748420, HQ917060, AY508697). (F) Alignment of loop motifs from EV-F sequence alignment of SELEX aptamers. Alignment of the 12 most frequently matched loop motifs reveals a preferred GAA motif. (G) N40 naïve library (grey) and EV-E selected library (red) matched against the PV Mahoney strain genome (NC_002058.3). Cognate PV SELEX peaks aligned against the Mahoney genome that are coincident with the EV-E peaks (inverted arrows for aptamers a42 in purple; a45 in orange; a50 in green). (H) Alignment of PV loop motifs, showing common AAR motif. (TIF) [file ppat.1009146.s002.tif]

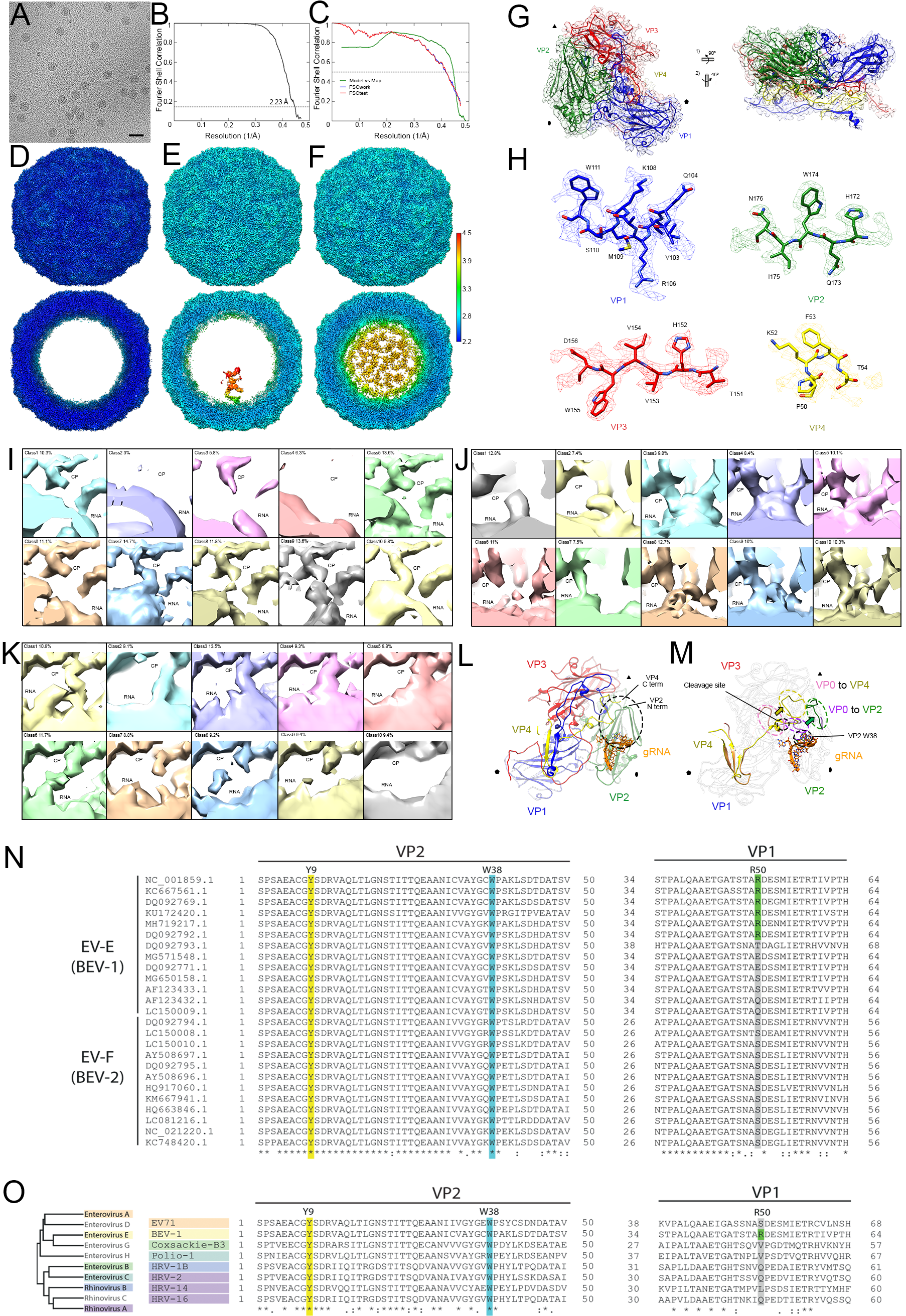

Supplement: S3 Fig — (A-F) Resolution and model validation of EV-E structure. (A) Cryo-EM image of EV-E. Bar = 500 Å. (B) Fourier Shell Correlation (FSC) resolution curve for the icosahedrally averaged 3DR of EV-E. Resolution based on the gold standard 0.143 criterion is 2.23 Å. (C) Cross-validation against overfitting of the model, FSC curve for the final atomic model refined against the post-processed map (green curve, Model vs Map), and FSC curves for the randomly shifted and refined atomic model against the half map used in the refinement (blue curve, FSCwork) and against the half map not used in the refinement (red curve, FSCtest). (D-F) Surface (top) and slab (bottom) viewed along a two-fold axis coloured and low-pass filtered based on local resolution as indicated in the colour key (values in Å) for the (D) icosahedrally averaged density map shown at 1 σ, (E) density map obtained after symmetry expansion and focused classification on two-fold axis shown at 2 σ, and (F) density map obtained after symmetry expansion and focused classification on genome density shown at 1.2 σ. (G-H) Quality of the cryoEM density map. G) Atomic model of the asymmetric unit of EV-E shown as ribbon diagrams (top view, left; side view, right) colour-coded as in Fig 2 fitted into the 2.2 Å resolution cryo-EM density map shown as colour-coded semi-transparent surface. Symbols indicate icosahedral symmetry axes. (H) Atomic models of EV-E viral proteins colour-coded and shown as sticks fitted into the 2.2 Å resolution cryo-EM density map shown as colour-coded mesh. Residues are indicated and coloured by heteroatom. (I) Variability of the VP2 W38-RNA contact. (I-K) Cryo-EM density maps for the 10 classes obtained after symmetry expansion and focused classification on two, three and five-fold axis shown at 2 σ and viewed as in Fig 3C, 3G and 3H, respectively. Class distribution, and CP and RNA densities are indicated. (L) EV-E VP0 processing. Atomic model of the asymmetric unit of EV-E shown as ribbon diagram [file ppat.1009146.s003.tif]

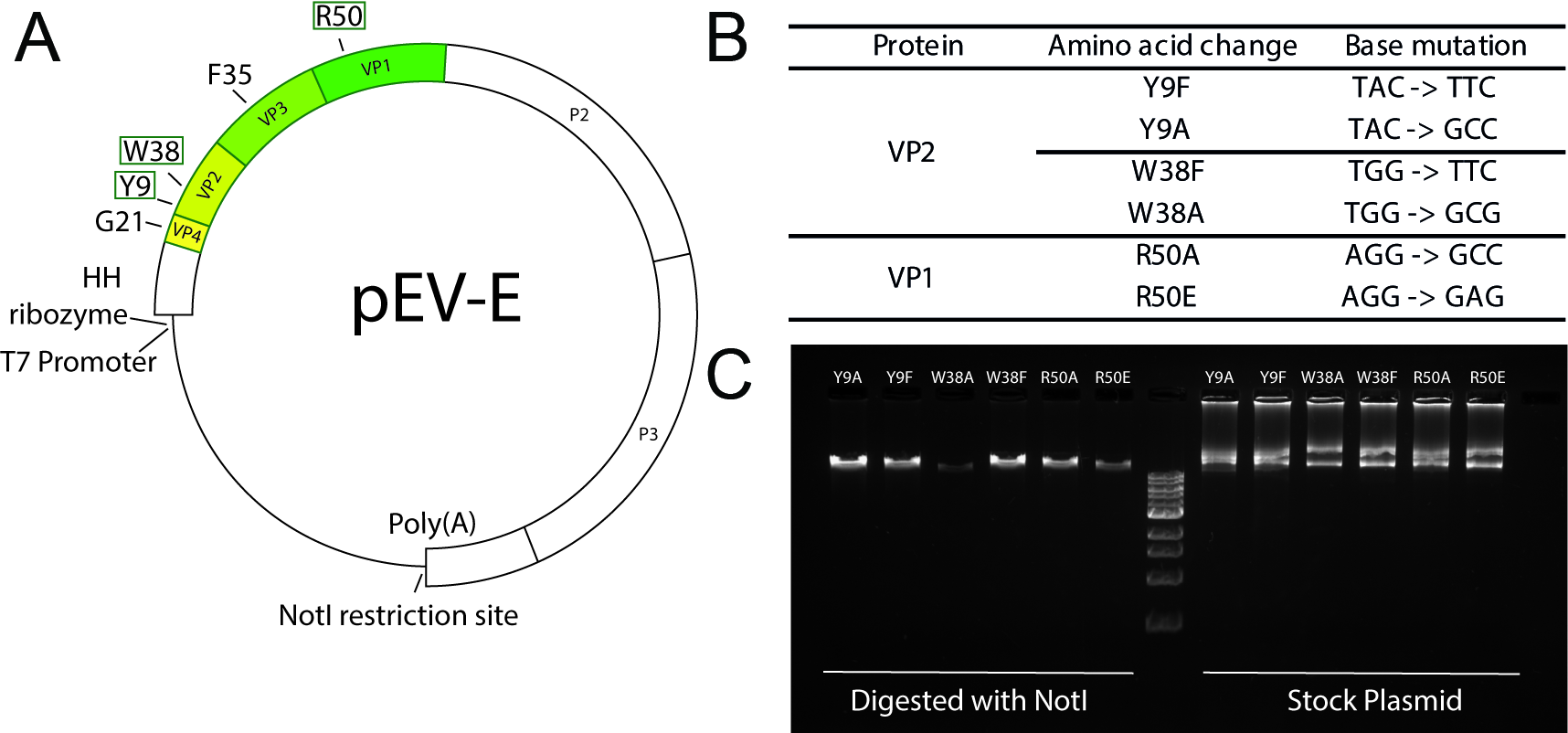

Supplement: S4 Fig — (A) Schematic of EV-E infectious clone; the EV-E genome was inserted into a pUC57 vector, the structural proteins of P1 region are filled in yellow-green and the sites of interest are highlighted, green boxes denote the chosen mutagenesis sites. (B) Mutations introduced individually into the infectious clone. (C) Plasmid digestion with Not I; cut and uncut plasmid were run on a 1%(w/v) agarose gel to confirm digestion. (TIF) [file ppat.1009146.s004.tif]
