# Supplementary material for: Assembly of infectious enteroviruses depends on multiple, conserved genomic RNA-coat protein contacts
Source: PLoS Pathog. 2020 Dec 28;16(12):e1009146. doi: 10.1371/journal.ppat.1009146 (PMC7793301; doi:10.1371/journal.ppat.1009146)
Supplement: S1 Table — (DOCX) [file ppat.1009146.s006.docx]

**Table S1: Cryo-EM structure.** Cryo-EM data collection, refinement and validation statistics.

|  | **EV-E sym I2**  **EMD-10504 / PDB: 6THD** | **EV-E sym exp + 2fold**  **EMD-10506 / PDB: 6THN** | **EV-E sym exp + genome**  **EMD-10505** |
| --- | --- | --- | --- |
| **Data collection and processing** |  |  |  |
| Microscope | FEI Titan Krios | FEI Titan Krios | FEI Titan Krios |
| Detector | FalconIII (linear mode) | FalconIII (linear mode) | FalconIII (linear mode) |
| Magnification | 75,000x | 75,000x | 75,000x |
| Voltage (kV) | 300 | 300 | 300 |
| Electron exposure (e^-^/Å^2^) | 49.5 | 49.5 | 49.5 |
| Exposure per frame (e^-^/Å^2^) | 1.27 | 1.27 | 1.27 |
| Defocus range (µm) | -0.75 to -3.5 | -0.75 to -3.5 | -0.75 to -3.5 |
| Pixel size (Å) | 1.065 | 1.065 | 1.065 |
| Micrographs collected (no.) | 8,785 | 8,785 | 8,785 |
| Initial particles (no.) | 260,348 | 105,723*60 = 6,343,380 | 105,723*60 = 6,343,380 |
| Final particles (no.) | 105,723 | 946,982 | 835,940 |
| Symmetry imposed | I2 | C1 | C1 |
| Map resolution (Å) | 2.23 | 2.60 | 2.62 |
| FSC threshold | 0.143 | 0.143 | 0.143 |
| Map resolution range (Å) | 2.20 – 2.67 | 2.50 – 4.90 | 2.50 – 4.13 |
| **Refinement** |  |  |  |
| Model resolution (Å) | 2.25 |  |  |
| FSC threshold | 0.5 |  |  |
| Mask correlation coefficient | 0.88 |  |  |
| Map sharpening B factor (Å^2^) | -75 |  |  |
| Model composition |  |  |  |
| Non-hydrogen atoms | 387,660 |  |  |
| Protein residues | 48,720 |  |  |
| Water | 5,820 |  |  |
| Ligands |  |  |  |
| MYR | 120 |  |  |
| SO_4_ | 120 |  |  |
| ADP (B-factors) min/max/mean |  |  |  |
| Protein | 3.18/41.17/8.52 |  |  |
| Water | 3.74/12.41/6.48 |  |  |
| Ligand | 6.13/29.33/16.80 |  |  |
| R.m.s. deviations |  |  |  |
| Bond lengths (Å) | 0.010 |  |  |
| Bond angles (˚) | 1.00 |  |  |
| Validation |  |  |  |
| MolProbity score | 1.60 |  |  |
| Clashscore | 5.71 |  |  |
| Rotamer outliers (%) | 0.29 |  |  |
| Ramachandran plot |  |  |  |
| Favored (%) | 95.85 |  |  |
| Allowed (%) | 4.03 |  |  |
| Outliers (%) | 0.12 |  |  |
